# Knowledge, attitudes, and practices toward tuberculosis among Jordanian university students

**DOI:** 10.3389/fpubh.2022.1055037

**Published:** 2022-11-21

**Authors:** Anas H. A. Abu-Humaidan, Alaa Tarazi, Yazan Hamadneh, Ahmad Al-leimon, Obada Al-leimon, Mohammad Aljahalin, Fatima Ahmad, Dima Awajan, Nader Alaridah

**Affiliations:** ^1^Department of Pathology, Microbiology and Forensic Medicine, School of Medicine, The University of Jordan, Amman, Jordan; ^2^School of Medicine, The University of Jordan, Amman, Jordan; ^3^Department of Clinical Pharmacy and Therapeutics, Applied Science Private University, Amman, Jordan

**Keywords:** tuberculosis, KAP, infectious diseases, university students, Jordan, Mtb (Mycobacterium tuberculosis), stigma and awareness, mask wearing

## Abstract

**Background:**

Tuberculosis (TB) is one of the leading causes of death from infectious diseases worldwide with numerous undiagnosed and untreated cases, emphasizing the need for TB awareness to minimize transmission and initiate early treatment. Data regarding the knowledge, attitudes, and practices (KAP) toward TB among Jordanians is lacking but requires attention given the massive migration spells to Jordan from neighboring countries in the past decade.

**Methods:**

A descriptive cross-sectional study was conducted from May to June 2022. An online questionnaire was developed following World Health Organization (WHO) recommendations for TB KAP surveys and was distributed to Jordanian university students. The questionnaire documented sociodemographic data and measured participants' KAP toward TB. Descriptive and analytic statistics were used to report KAP levels and highlight relevant sociodemographic factors associated with better KAP.

**Results:**

602 participants completed the survey; most were females (60.8%), in their first 3 years of school (84.4%), and from a healthcare field of study (57.0%). The knowledge section median score was 27 out of 51. Knowledge gaps in TB treatment, and to a lesser extent, TB transmission routes were identified. The attitudes section median score was 6 out of 9, attitudes were generally positive toward TB patients with no indication of a social stigma. The practice section median score was 6 out of 8, most participants would take the correct measures if they suspected being infected, yet around 41.0% were not confident that masks are important in preventing airborne diseases. Students in healthcare specialties had significantly better KAP scores and identifying as a smoker was associated with a lower practice score.

**Conclusion:**

Although university students displayed satisfactory KAP scores, the focus should be aimed at informing students from non-healthcare fields on TB transmission routes, treatment options, and the role of masks in preventing disease transmission.

## Introduction

Tuberculosis (TB) is a communicable disease caused by the bacillus *Mycobacterium tuberculosis* (Mtb), which is spread by aerosols expelled from people with active TB disease. Right until the coronavirus (COVID-19) pandemic, TB was the leading cause of death from a single infectious agent, ranking above HIV/AIDS as indicated by the World Health Organization (WHO) Global tuberculosis report 2021 (1). Infection with Mtb occurs primarily by aerosolized particles when coughing or sneezing, ~90% of those who develop the disease are adults, with more cases among men than women. Pulmonary TB causes symptoms such as persistent cough with sputum and blood at times, chest pain, weakness, weight loss, fever, and night sweats. Other less recognized extrapulmonary infection sites are the skeletal and central nervous systems (2).

Jordan is considered one of the low-burden countries with a total TB incidence below 10 cases per 100,000 in 2019 (3), however, its location near several war zones led to the country receiving around 760,000 refugees and asylum seekers registered with UNHCR over the past decade (4). Previous studies of such crisis-affected populations found higher TB incidence and delayed TB treatment compared with reference populations (5).

The arrival of Syrian refugees led to a considerable impact on Jordan's national TB program, where TB cases among Syrian refugees made up 24.4 and 13.8% of all TB cases in Jordan in 2013 and 2015, respectively (6). Since refugees are increasingly having access to higher education (7), it is likely that university students, who form a considerable percentage of Jordanian society, will be in contact with displaced and resettled populations through shared education, volunteer work, or proximity in residence, and in the case of healthcare specialties, when they join the workforce. Additionally, funding in low- and middle-income countries (LMIC), which account for 98% of reported TB cases, cannot cope with the increased TB burden (1).

Most of the studies regarding knowledge and attitude toward TB were done in high-burden TB countries such as India (8), Uganda (9), and South Africa (10). Previous KAP surveys were performed in neighboring countries but not in Jordan and did not include non-healthcare university students (11). University students, whether in the healthcare field or not, are an important subgroup of the population whose decisions could impact transmission trends in the future, hence several studies investigated KAP toward TB in this specific group (12–14).

Given the increased TB burden in Jordan associated with refugees' resettlement and the importance of university students in determining the future of TB burden in the country, this cross-sectional study aimed to measure KAP toward TB among university students in Jordan using an online questionnaire adopted from a WHO guide to developing TB KAP surveys (15).

## Materials and methods

### Study design and population

This was a descriptive cross-sectional study. The sample consisted of students from all schools enrolled at 6 Jordanian universities (Supplementary Figure 1). These Jordanian Universities have around 206,000 students enrolled in various programs and schools (16). Data were collected from healthcare schools (e.g., Medicine, Dentistry, Pharmacy, and Nursing), and non-healthcare schools (e.g., Physics, Mechanical engineering, Law, Business, and Arts).

The study was conducted using an online questionnaire created on Google Forms and delivered to students in the period between 22 May and 23 June 2022. The questionnaire was posted on online groups that the students use for school-specific communications, student communication channels are moderated by student committees who grant access only to students with verified university email addresses. In addition, representatives from each university were contacted to help in distributing the questionnaire.

### Questionnaire design

This questionnaire was based on WHO recommendations for TB KAP surveys with a slight modification (15), which involved removing detailed questions regarding HIV/AIDS since it is not prevalent in Jordan. The first page of the questionnaire included an informed consent form, as well as a description of the contents of the questionnaire, what it aims to study, and the confidentiality of the data. It was written in both Arabic and English. To assess the accuracy of the questionnaire, an internal pre-validation procedure was carried out at all 6 Jordanian universities involving 38 students and was re-evaluated with input from experts in the field.

The questionnaire consisted of 35 multiple-choice questions that covered four main themes: (1) Demographics and general information about the participant (sex, age, field of study, academic year, smoking, Nationality, and residence), (2) Knowledge of TB symptoms, transmission routes, at-risk groups, and treatment. (3) TB attitudes, practices, and care-seeking behavior, (4) TB awareness and sources of information.

Questions were scored as follows: for questions with a single correct answer, correct and incorrect answers were scored as 5 and 0, respectively. While for questions with multiple correct answers, correct and incorrect answers were scored as 1 and 0, respectively. This scoring system was done to equalize the weight given for each knowledge question, whether it had one, or more than one correct answer. The internal consistency of the questionnaire was measured using Cronbach's alpha. The questionnaire can be found in the Supplementary material.

### Ethical approval

The study protocol was approved by the Institutional Review Board (IRB) at UJ (Ref. No. 19/2022/271). Decision No. (46-2022). In addition, the work was conducted according to the principles of Good Clinical Practice (GCP) that has its origin in the Declaration of Helsinki (64th World Medical Association General Assembly, Fortaleza, Brazil, October 2013). All collected data were treated with confidentiality. Participation in the study was voluntary. Informed consent was obtained from all participants at the start of the questionnaire following a full explanation of the study objectives, then they were asked to complete an anonymous questionnaire.

### Data analysis

Data generated were organized in Microsoft Excel, and statistical analysis was carried out using IBM Statistical Package for the Social Sciences (SPSS) for Windows version 25.0 (Armonk, NY, USA) and GraphPad Prism 8 (San Diego, CA, USA).

Categorical variables were presented as count and percentages in the tables and as percentages in the text, while continuous variables were presented as (Mean ± SD). Independent sample *T*-tests, one-way ANOVA, and Chi-square test were used to compare KAPS between different school categories or demographic variables. A *p*-value of 0.05 was adopted as a threshold for significance.

## Results

### Demographics and characteristics of participants

Six hundred and two (602) students completed the survey. Students were from six Jordanian universities that are distributed among the major governorates of Jordan (Supplementary Figure 1). Males represented 39.2% of the sample, while females represented 60.8%. The median age of participants was 20 years with 402 participants (66.8%) between 18 and 21 years old and 200 participants (33.2%) between 22 and 26 years old. Most (36.5%) participants were in their second year of study. The majority (78.2%) were cities/central urban areas residents, whilst 21.8% resided in rural and countryside areas. The participants were grouped by their field of study as healthcare students (57%) from fields such as medicine, nursing, and pharmacy, or non-healthcare students (43%) from fields such as law, engineering, and arts. Also, most (79.1%) participants described themselves as non-smokers and only 10% knew someone who had TB (Table 1).

**Table 1:** Participants' demographics.

**Demographic variable**	**Count (%)**
**Gender**	
Male	236 (39.2%)
Female	366 (60.8%)
**Age group**	
18–21	402 (66.8%)
22–26	200 (33.2%)
**Year of study**	
First	163 (27.1%)
Second	220 (36.5%)
Third	125 (20.8%)
Fourth	73 (12.1%)
Fifth	17 (2.8%)
Sixth	4 (0.7%)
**Field of study**	
Healthcare field	343 (57%)
Non-healthcare field	259 (43%)
**Smoking**	
Yes	126 (20.9%)
No	476 (79.1%)
**Residence**	
Urban	471 (78.2%)
Rural	131 (21.8%)
**Knew someone with TB**	
Yes	60 (10%)
No	542 (90%)

### Knowledge of TB among university students

Knowledge of TB was assessed through questions regarding symptoms, transmission routes, high-risk groups, and treatment. As for symptoms, most participants (75.4%) identified “coughing up blood” as a symptom of TB, this was followed by 62.8% of participants who chose “cough that lasts for more than 3 weeks” which is the most reported TB symptom, indicating that most participants have good knowledge about the symptoms of TB. Still, 16.1% of participants stated that they did not know any of the symptoms. When asked about spinal tuberculosis or a relationship between bone inflammation and tuberculosis, most participants (51.8%) were not sure if such a relation exists (Table 2).

**Table 2:** Knowledge of TB symptoms, transmission routes, and risk groups^a^.

**Question**	**Answer^b^**	**All students** **(*n* = 602)**	**Healthcare students (*n* = 343)**	**Non-healthcare students (*n* = 259)**
What are the signs and symptoms of TB?^c^	Cough that lasts for more than 3 weeks	378 (62.8%)	249 (72.6%)	129 (49.8%)
	**Coughing up blood**	**454 (75.4%)**	**283 (82.5%)**	**171 (66.0%)**
	Severe headache	134 (22.3%)	79 (23.0%)	55 (21.2%)
	Weight loss	251 (41.7%)	183 (53.4%)	68 (26.3%)
	Fever	304 (50.5%)	213 (62.1%)	91 (35.1%)
	Chest pain	300 (49.8%)	211 (61.5%)	89 (34.4%)
	Shortness of breath	323 (53.7%)	227 (66.2%)	96 (37.1%)
	Ongoing fatigue	294 (48.8%)	210 (61.2%)	84 (32.4%)
	I do not know	97 (16.1%)	34 (9.9%)	63 (24.3%)
How can a person get TB?^c^	Through handshakes	135 (22.4%)	86 (25.1%)	49 (18.9%)
	**Through the air when the person with TB coughs or sneezes**	**401 (66.6%)**	**272 (79.3%)**	**129 (49.8%)**
	Through sharing dishes	185 (30.7%)	113 (32.9%)	72 (27.8%)
	Through eating from the same plate	9 (1.5%)	6 (1.7%)	3 (1.2%)
	Through touching items in public spaces (doorknobs, handles in transportation, etc)	184 (30.6%)	113 (32.9%)	71 (27.4%)
	It is not a contagious disease	38 (6.3%)	17 (5.0%)	21 (8.1%)
	I do not know	143 (23.8%)	44 (12.8%)	99 (38.2%)
In your opinion, who can be infected with TB?^c^	**Anybody**	**417 (69.3%)**	**246 (71.7%)**	**171 (66.0%)**
	Poor people	79 (13.1%)	64 (18.7%)	15 (5.8%)
	Homeless people	86 (14.3%)	68 (19.8%)	18 (6.9%)
	Alcoholics	77 (12.8%)	60 (17.5%)	17 (6.6%)
	Drug users	86 (14.3%)	65 (19.0%)	21 (8.1%)
	People living with HIV or AIDS	184 (30.6%)	140 (40.8%)	44 (17.0%)
	People who have been in prison	81 (13.5%)	63 (18.4%)	18 (6.9%)
	I do not know	94 (15.6%)	33 (9.6%)	61 (23.6%)
Do you think that there is a relationship between TB and bone inflammation or curvature of the spine?^c^	Yes	163 (27.1%)	116 (33.8%)	47 (18.1%)
	No	127 (21.1%)	66 (19.2%)	61 (23.6%)
	**Maybe**	**312 (51.8%)**	**161 (46.9%)**	**151 (58.3%)**
Do you think that immunocompromised people are more susceptible to TB?^c^	**Yes**	**526 (87.4%)**	**319 (93.0%)**	**207 (79.9%)**
	No	17 (2.8%)	7 (2.0%)	10 (3.9%)
	I do not know	59 (9.8%)	17 (5.0%)	42 (16.2%)

a Data is presented as count and percent.

b Most chosen answers are in bold.

c Answers to these questions differed significantly between healthcare and non-healthcare students.

Participants were also asked about the routes of transmission of the disease, and most participants (66.6%) selected the statement “Through the air when the person with TB coughs or sneezes,” which is thought to be the most common way of TB transmission. A small proportion (6.3%) thought it was not a contagious disease. Notably, around one-quarter (23.8%) did not know transmission routes (Table 2).

When asked about populations at risk, only a minority of participants (10–15%) correctly chose the various risk groups, except for the group “people living with HIV or AIDS” which was chosen by 30.6% of participants. Additionally, most participants correctly identified that immunocompromised participants (87.4%) are more susceptible to TB (Table 2).

When asked about TB treatment, almost half (49.5%) of the participants agreed that TB can be cured, with almost the other half (44.4%) not sure if it can be cured or not. Concerning side effects, 37.2% of participants thought that it can have minor side effects, while the most chosen answer was “I do not know” (45.8%). Also, most participants (52.2%) did not know the duration of TB treatment, and only 27.4% correctly answered that it needs 6–12 months. As for TB vaccines, most (79.4%) participants agree that there is a vaccine that can protect against TB (Table 3).

**Table 3:** Knowledge of TB treatment and vaccines^a^.

**Question**	**Answer^b^**	**All students** **(*n* = 602)**	**Healthcare students (*n* = 343)**	**Non-healthcare students (*n* = 259)**
Can TB be cured?^c^	**Yes**	**298 (49.5%)**	**200 (58.3%)**	98 (37.8%)
	No	37 (6.1%)	15 (4.4%)	22 (8.5%)
	Maybe	267 (44.4%)	128 (37.3%)	**139 (53.7%)**
How can someone with TB be cured?^c^	Herbal remedies	54 (9%)	17 (5.0%)	37 (14.3%)
	Home rest without medicine	17 (2.8%)	8 (2.3%)	9 (3.5%)
	**Specific drugs provided by a health center or a doctor**	**470 (78.1%)**	**300 (87.5%)**	**170 (65.6%)**
	I do not know	128 (21.3%)	46 (13.4%)	82 (31.7%)
How long does it take to treat TB?^c^	<6 Months	66 (11%)	34 (9.9%)	32 (12.4%)
	6–12 months	165 (27.4%)	137 (39.9%)	28 (10.8%)
	13–18 months	13 (2.2%)	5 (1.5%)	8 (3.1%)
	19–24 months	18 (3%)	10 (2.9%)	8 (3.1%)
	More than 24 months	26 (4.3%)	16 (4.7%)	10 (3.9%)
	**I do not know**	**314 (52.2%)**	**141 (41.1%)**	**173 (66.8%)**
Does TB treatment have side effects?^c^	It does not have side effects	12 (2%)	6 (1.7%)	6 (2.3%)
	It can have minor side effects (e.g., nausea, anorexia, joint pain, orange red urine)	224 (37.2%)	**146 (42.6)**	78 (30.1%)
	It can have severe side effects (e.g., deafness, vomiting, jaundice, visual impairment)	90 (15%)	60 (17.5%)	30 (11.6%)
	**I do not know**	**276 (45.8%)**	131 (38.2%)	**145 (56.0%)**
Do we have vaccines that can protect us against TB?	**Yes**	**478 (79.4%)**	**276 (80.5%)**	**202 (78.0%)**
	No	124 (20.6%)	67 (19.5%)	57 (22.0%)

a Data is presented as count and percent.

b Most chosen answers are in bold.

c Answers to these questions differed significantly between healthcare and non-healthcare students.

The most chosen answers to knowledge questions were rather similar in healthcare and non-healthcare student groups, but the distribution of correct answers varied significantly in most questions (Table 2). Non-healthcare students tended to choose (maybe) and (I do not know) more often than healthcare students especially with regards to TB treatment. For example, 53.7% of non-healthcare students were not sure whether TB can be treated or not compared to 37.3% of healthcare students, similar results were also found with regards to TB treatment side effects (Table 3). The only question with similar answers in both groups was about the availability of vaccines that protect from TB, to which 80.5 and 78.0% of healthcare and non-healthcare students, respectively, answered in the affirmative (Table 3).

### Attitudes and practices toward TB

In the attitude section, participants were first asked whether they believe they can get TB, to which (62.5%) of participants believed they could. Afterward, participants were asked how they view, and how they think society views, TB patients. Most participants felt compassion and a desire to help (41.7%) or compassion but would rather stay away (30.9%) from TB patients. Similarly, most (52.0%) thought their communities are friendly but would stay away from TB patients. As for the TB situation in Jordan, 45.3% of participants thought it was somewhat serious, and only 11.5% knew that TB treatment in Jordan was free of charge (Table 4).

**Table 4:** Attitudes toward TB^a^.

**Question**	**Answer^b^**	**All students** **(*n* = 602)**	**Healthcare students (*n* = 343)**	**Non-healthcare students (*n* = 259)**
Do you think you can get TB?	**Yes**	**376 (62.5%)**	**214 (62.4%)**	**162 (62.5%)**
	No	226 (37.5%)	129 (37.6%)	97 (37.5%)
Which statement is closest to your feeling about people with TB disease?	**I feel compassion and a desire to help**	**251 (41.7%)**	**147 (42.9%)**	**104 (40.2%)**
	I feel compassion but I tend to stay away from these people.	186 (30.9%)	109 (31.8%)	77 (29.7%)
	It is their problem and I cannot get TB	4 (0.7%)	3 (0.9%)	1 (0.4%)
	I fear them because they may infect me	29 (4.8%)	12 (3.5%)	17 (6.6%)
	I have no particular feeling	132 (21.9%)	72 (21.0%)	60 (23.2%)
In your community, how is a person who has TB usually regarded/treated?	Most people reject him or her	165 (27.4%)	88 (25.7%)	77 (29.7%)
	**Most people are friendly, but they generally try to avoid him or her**	**313 (52.0%)**	**199 (58.0%)**	**114 (44.0%)**
	The community mostly supports and helps him or her	124 (20.6%)	56 (16.3%)	68 (26.3%)
How serious a problem do you think TB is in Jordan?^c^	Very serious	121 (20.1%)	60 (17.5%)	61 (23.6%)
	**Somewhat Serious**	**273 (45.3%)**	**168 (49.0%)**	**105 (40.5%)**
	Not serious at all	50 (8.3%)	36 (10.5%)	14 (5.4%)
	I don't know	158 (26.2%)	79 (23.0%)	79 (30.5%)
How expensive do you think TB diagnosis and treatment is in Jordan?	It is free of charge	68 (11.5%)	35 (10.2%)	33 (12.7%)
	It is reasonably priced	76 (12.8%)	46 (13.4%)	30 (11.6%)
	It is somewhat/moderately expensive	141 (23.8%)	87 (25.4%)	54 (20.8%)
	It is very expensive	41 (6.9%)	28 (8.2%)	13 (5.0%)
	**I don't know**	**267 (45%)**	**145 (42.3%)**	**122 (47.1%)**

a Data is presented as count and percent.

b Most chosen answers are in bold.

c Answers to these questions differed significantly between healthcare and non-healthcare students.

Health-seeking behavior was assessed as well. Participants were asked about their actions if they had a persistent or bloody cough and most participants (87.9%) choose to go to the doctor over other options like drinking herbs, taking any medicine from the pharmacy, or just ignoring it. To expand on this point, participants were asked about when they might go to a health facility if they had TB symptoms, most (68.4%) would go as soon as they realize that their symptoms might be related to TB, indicating positive health-seeking behaviors in most participants (Table 5).

**Table 5:** Practices related to TB and respiratory diseases among university students^a^.

**Question**	**Answer^b^**	**All students** **(*n* = 602)**	**Healthcare students (*n* = 343)**	**Non-healthcare students (*n* = 259)**
If you had a cough for more than three weeks or if you were coughing up blood in your sputum, what would you do?^c^	**Go to a doctor**	**529 (87.9%)**	**317 (92.4%)**	**212 (81.9%)**
	Drink herbs	20 (3.3%)	4 (1.2%)	16 (6.2%)
	Take any medicine from the pharmacy	27 (4.5%)	9 (2.6%)	18 (6.9%)
	Just Ignore it	26 (4.3%)	13 (3.8%)	13 (5.0%)
Who would you talk to about your illness if you had TB?^c^	**A doctor or any healthcare provider**	**493 (81.9%)**	**314 (91.5%)**	**179 (69.1%)**
	Spouse	83 (13.8%)	58 (16.9%)	25 (9.7%)
	Parent	326 (54.2%)	193 (56.3%)	133 (51.4%)
	Children	36 (6%)	22 (6.4%)	14 (5.4%)
	Other family members	178 (29.5%)	98 (28.6%)	80 (30.9%)
	Close friends	160 (26.6%)	100 (29.2%)	60 (23.2%)
	Others	42 (7%)	23 (6.7%)	19 (7.3%)
	No one	39 (6.5%)	14 (4.1%)	25 (9.7%)
If you had symptoms of TB, at what point would you go to the health facility?^c^	When treatment on my own does not work	47 (7.8%)	14 (4.1%)	33 (12.7%)
	When symptoms that look like TB signs last for 3–4 weeks	134 (22.3%)	79 (23.0%)	55 (21.2%)
	**As soon as I realize that my symptoms might be related to TB**	**412 (68.4%)**	**248 (72.3%)**	**164 (63.3%)**
	I would not go to the doctor	9 (1.5%)	2 (0.6)	7 (2.7%)
If you would not go to the health facility, what is the reason?	**Not sure where to go**	**4 (44.4%)**	**1 (0.3%)**	**3 (1.2%)**
	Cost and difficulties with transportation/distance to clinic	2 (22.2%)	**1 (0.3%)**	1 (0.4%)
	Do not trust medical workers	0 (0%)	0 (0.0%)	0 (0.0%)
	Do not like attitude of medical workers	1 (11.1%)	0 (0.0%)	1 (0.4%)
	Cannot leave work (overlapping work hours with medical facility working hours)	0 (0%)	0 (0.0%)	0 (0.0%)
	Do not want to find out that something is really wrong.	2 (22.2%)	0 (0.0%)	2 (0.8%)
Do you use hygiene products in public places?	Yes	241 (40%)	141 (41.1%)	100 (38.6%)
	No	97 (16.1%)	53 (15.5%)	45 (17.4%)
	**Sometimes**	**264 (43.9%)**	**149 (43.4%)**	**114 (44.0%)**
Are you convinced that the mask is an appropriate way to prevent infectious diseases that can be transmitted in air?^c^	**Yes**	**355 (59%)**	**232 (67.6%)**	**123 (47.5%)**
	No	96 (15.9%)	39 (11.4%)	57 (22.0%)
	Maybe	151 (25.1%)	72 (21.0%)	79 (30.5%)
Were your daily habits related to the transmission of chest diseases affected by the COVID-19 pandemic?	**Yes**	**463 (76.9%)**	**268 (78.1%)**	**195 (75.3%)**
	No	139 (23.1%)	75 (21.9%)	64 (24.7%)

a Data is presented as count and percent.

b Most chosen answers are in bold.

c Answers to these questions differed significantly between healthcare and non-healthcare students.

This behavior was further emphasized by most participants (81.9%) choosing their doctor or health provider to talk to about their symptoms. A good percent of participants also chose their parents, other family members, or close friends, (54.2, 29.5, and 26.6%, respectively) to tell them about their illness, signaling trust in medical institutes as well as family members, with little signs of stigma around the disease (Table 5).

As for hygiene practices, most participants indicated they sometimes use hygiene products in public (43.9%), other participants use them always (40.0%), and 16.1% don't use them at all. Notably, a large proportion of participants either were not sure (25.1%) or did not believe (15.9%) that masks can prevent the transmission of airborne infectious diseases. Most participants believed that their daily habits related to the transmission of respiratory diseases were affected by the COVID-19 pandemic (76.9%) (Table 5).

Unlike the significant differences in answers to knowledge questions, attitudes were rather similar in healthcare and non-healthcare student groups, which were generally positive toward TB patients (Table 4). On the other hand, practices in the two groups differed in important aspects, such as the person to talk to if the participant had TB, where around 91.5 and 69.1% of healthcare and non-healthcare students, respectively, chose a doctor or a healthcare provider, this indicated that almost one third of non-healthcare students would not choose to talk about their disease with a doctor (Table 5). Another significant difference was found with regards to wearing a mask to prevent infections transmitted by air, where more than half of non-healthcare students (52.5%) were not convinced that wearing a mask might be useful, in contrast to only (32.4%) of healthcare students (Table 5).

### Characteristics associated with better KAP

Participants were grouped according to the characteristics mentioned in Table 1, and the average KAP score was compared among those groups using appropriate statistical tests. There was a significant difference in the knowledge score, but not attitude and practice scores according to the year of study, but there was no incremental knowledge score with more years of study (Table 6). Expectedly, students in the healthcare field had significantly better knowledge scores compared to non-healthcare students (30.8 ± 10.1 vs. 21.6 ± 9.8, respectively, *p* < 0.001), which was also reproduced in attitude scores (6.2 ± 1.7 vs. 5.9 ± 1.7, respectively, *p* = 0.027) and practice scores (6.4 ± 1.3 vs. 5.6 ± 1.6, respectively, *p* < 0.001) (Table 6).

**Table 6:** Demographic variables associated with better TB KAPS^a^.

**Demographic variable**	**Mean knowledge score (±SD)**	***P*-value**	**Mean attitude score (±SD)**	***P*-value**	**Mean practice score (±SD)**	***P*-value**
**Year of study**						
First	23.4 (±8.8)	**<0.001**	6.2 (±1.6)	0.511	5.8 (±1.4)	0.402
Second	29.2 (±10.9)		6.1 (±1.8)		6.1 (±1.5)	
Third	27.9 (±11.1)		6.1 (±1.8)		6.1 (±1.7)	
Fourth	25.6 (±12.4)		5.8 (±1.4)		6.1 (±1.4)	
Fifth	26.9 (±14.4)		5.8 (±2.1)		6.4 (±1.2)	
Sixth	28.3 (±13.2)		5.0 (±1.6)		5.8 (±1.5)	
**Gender**						
Male	27.6 (±11.4)	0.157	6.2 (±1.7)	0.065	5.9 (±1.5)	0.115
Female	26.3 (±10.7)		6.0 (±1.7)		6.1 (±1.5)	
**Field of study**						
Healthcare field	30.8 (±10.1)	**<0.001**	6.2 (±1.7)	**0.027**	6.4 (±1.3)	**<0.001**
Non-healthcare field	21.6 (±9.8)		5.9 (±1.7)		5.6 (±1.6)	
**Smoking**						
Yes	25.2 (±11.9)	0.065	5.8 (±1.8)	0.099	5.6 (±1.6)	**<0.001**
No	27.3 (±10.7)		6.1 (±1.7)		6.2 (±1.5)	
**Residence**						
Urban	27.3 (±11.2)	0.088	6.1 (±1.7)	0.623	6.0 (±1.5)	0.930
Rural	25.4 (±10.0)		6.1 (±1.8)		6.0 (±1.5)	
**Knew someone with TB**						
Yes	27.5 (±10.6)	0.617	6.2 (±1.7)	0.468	5.6 (±1.7)	**0.026**
No	26.8 (±11.0)		6.1 (±1.7)		6.1 (±1.5)	

a Statistically significant differences are in bold.

Participants who knew someone with TB did not show a significant difference in knowledge and attitude scores compared to those who did not know someone with TB, but there was a significant difference in the practice score, which was surprisingly better in those who did not know a person with TB (5.6 ± 1.7 vs. 6.1 ± 1.5, *p* = 0.026) (Table 6). There was a better practice score among non-smokers compared to smokers (6.2 ± 1.5 vs. 5.6 ± 1.6, respectively, *p* < 0.001), but no differences were found in attitude or knowledge scores. Finally, there was no significant difference in KAP scores when comparing males to females, or urban to rural residents.

The correlations between knowledge and attitude scores, as well as knowledge and practice scores, were described using Spearman's correlation coefficient (*r*_*s*_). There was a weak positive but significant correlation between Knowledge and attitude scores (*r*_*s*_ = 0.243, 95% CI 0.164–0.319, *p* < 0.001) as well as between knowledge and practice scores (*r*_*s*_ = 0.314, 95% CI 0.237–0.386, *p* < 0.001) (Supplementary Figure 2).

### TB awareness and sources of information

Participants were asked about the first time they learned about TB. Most learned about TB for the first time from TV (38.4%), with school or college and social media coming in second and third places respectively. Two other close choices were family or friends and teachers (22.4 and 21.6%, respectively). Only 7.3% of participants didn't hear about TB before. Most participants (73.6%) did not feel that they were well informed about TB, and this was reflected in the wish of most participants (85.9%) to learn more about TB (Table 7).

**Table 7:** Sources of information used to learn about TB^a^.

**Question**	**Answer^b^**	**All students** **(*n* = 602)**	**Healthcare students (*n* = 343)**	**Non-healthcare students (*n* = 259)**
Where did you first learn about TB?^c^	Newspapers and magazines	52 (8.6%)	26 (7.6%)	26 (10.0%)
	Social media	180 (29.9%)	85 (24.8%)	95 (36.7%)
	Teachers	130 (21.6%)	69 (20.1%)	61 (23.6%)
	**Television**	**231 (38.4%)**	128 (37.3%)	**103 (39.8%)**
	Brochures, posters, and other printed materials	68 (11.3%)	34 (9.9%)	34 (13.1%)
	Healthcare workers	59 (9.8%)	36 (10.5%)	23 (8.9%)
	Family and friends	135 (22.4%)	65 (19.0%)	70 (27.0%)
	School or college	188 (31.2%)	**178 (51.9%)**	10 (3.9%)
	Never heard about it	44 (7.3%)	14 (4.1%)	30 (11.6%)
Do you feel well informed about TB?^c^	Yes	159 (26.4%)	122 (35.6%)	37 (14.3%)
	**No**	**443 (73.6%)**	**221 (64.4%)**	**222 (85.7%)**
Do you wish you could get more information about TB?	**Yes**	**517 (85.9%)**	**301 (87.8%)**	**216 (83.4%)**
	No	85 (14.1%)	42 (12.2%)	43 (16.6%)
What are the sources of information that you think can most effectively reach people like you with information on TB?	Newspapers and magazines	78 (13%)	46 (13.4%)	32 (12.4%)
	**Social media**	**477 (79.2%)**	**269 (78.4%)**	**208 (80.3%)**
	Radio	54 (9%)	20 (5.8%)	34 (13.1%)
	Television	195 (32.4%)	102 (29.7%)	93 (35.9%)
	Billboards	147 (24.4%)	76 (22.2%)	71 (27.4%)
	Brochures, posters, and other printed materials	125 (20.8%)	66 (19.2%)	59 (22.8%)
	Healthcare workers	315 (52.3%)	221 (64.4%)	94 (36.3%)
	Family and friends	205 (34.1%)	112 (32.7%)	93 (35.9%)
	Teachers	231 (38.4%)	137 (39.9%)	94 (36.3%)

a Data is presented as count and percent.

b Most chosen answers are in bold.

c Answers to these questions differed significantly between healthcare and non-healthcare students.

At the end of the questionnaire, participants were asked about the source they thought was most effective for TB education with 9 possible choices: newspapers and magazines, social media, radio, TV, billboards, brochures or posters, and other printed materials, healthcare workers, family and friends, and teachers. The top two sources chosen were social media (79.2%) and healthcare workers (52.3%) (Table 7).

It should be noted that healthcare and non-healthcare student groups varied significantly in terms of where they first learned about TB, since most healthcare students (51.9%) chose school or college compared to only 3.9% of non-healthcare students. Additionally, 85.7% of non-healthcare students felt they were not well informed about TB, compared to 64.4% of healthcare students. But both groups wanted to be more informed about TB and chose social media as the preferred source of information with no significant differences between the two (Table 7).

## Discussion

While similar KAP surveys have been carried out in neighboring countries (17, 18), this descriptive cross-sectional study was the first to measure KAP toward TB among university students in Jordan. Although KAP scores were satisfactory, this study revealed knowledge gaps related to TB transmission and treatment especially in non-healthcare students, additionally, it showed no social stigma around TB, good health seeking behavior, and doubt regarding the use of masks in preventing respiratory disease.

Awareness of infectious respiratory diseases such as TB is considered the first step in reducing their burden on the community. KAP surveys should draw attention to knowledge gaps, disease stigma, or practices that increase the chance of disease transmission. Since KAP surveys usually yield distinct results amongst various communities around the world (19–21), it is essential to conduct them in different settings to form targeted awareness campaigns and specific regional policies.

The sample that was studied was representative of Jordanian university students to a certain extent, firstly, females represent the larger proportion of university students in Jordan, which explains their representation in this study (16), secondly, the universities that were included in this study are the largest and most diverse in Jordan since they host students from different parts of the country (16). On the other hand, healthcare students were overrepresented in this study, probably since they are more interested in health-related topics and are more willing to complete such surveys, which denotes a selection bias. Nevertheless, the almost equal number of students from healthcare and non-healthcare-related fields in this study allowed for a more accurate comparison between the two groups.

Total knowledge scores in this study were satisfactory, and when compared to a national survey performed among the general public in Nigeria, which is a high-burden TB country, better knowledge scores were found in this study, but the authors of the study noted that respondents with tertiary education had the highest TB knowledge scores, comparable to scores in this study (22), which highlights the need for TB educational programs for those who do not attend higher education.

Specific knowledge weaknesses were identified in this study, especially in knowledge about TB treatment, where almost half of the respondents were not sure if TB can be cured and one-fifth did not know of the presence of vaccines, although the Bacille Calmette Guerin (BCG) vaccine is part of the mandatory vaccination program in Jordan. Moreover, about a quarter of the participants did not know about TB routes of transmission and about 16% did not know any of the symptoms, which is a considerable percentage given the educational level of the population being studied. This issue was further highlighted when examining answers from non-healthcare students separately, where over one third of students (38.2%) did not know TB routes of transmission and around a quarter did not know any TB symptoms or the people at risk of TB. This should be addressed by incorporating TB knowledge in health curricula given to non-healthcare specialties in Jordanian universities. The benefit of introducing general TB knowledge to non-healthcare students at university is supported by the fact that only 3.9% of non-healthcare students learned about TB from school or college, compared to 51.9% of healthcare students.

Students in healthcare fields showed significantly better KAP scores than students from non-healthcare fields, which is unsurprising given their training and has been demonstrated in other related TB knowledge surveys (23). But unlike studies that found KAP levels to be associated with gender or age (24, 25), this study found a better KAP score only in healthcare students and a worse practice score in smokers.

KAP surveys completed by students from healthcare fields in Jordan usually reveal satisfactory scores, as demonstrated by a recent study that measured KAP toward COVID-19 (26). Similarly, a cross-sectional study on Italian healthcare students showed satisfactory TB knowledge, but concluded that there could be places for improvement in healthcare curricula, especially for nursing students (27). Another study on Iranian medical students showed good KAP scores but found gaps in TB transmission and diagnosis knowledge (28). In contrast, healthcare students in this study displayed good knowledge in transmission routes with around 80% correctly identifying the most common route of transmission. It should be noted that the questionnaire used in this study was different from the two studies mentioned earlier as it was not directed solely to healthcare students, thus the questions did not require in-depth knowledge with regards to treatment and diagnosis. Rather, this survey was based on WHO recommendations for TB KAP surveys with some modifications made to fit the population being tested (15).

Before the recent migration spells from Syria, a community-based study investigated the prevalence of TB suspects (defined as persons with a persistent cough for more than 3 weeks) in Jordan in 2008 and reported a relatively low prevalence of 2.51% (29). Interestingly, the study also investigated attitudes of TB suspects and showed a high level of stigma, in contrast to what we found in this study, since >70% of participants indicated they feel compassion for TB patients, and >70% thought that the community is usually friendly to TB patients. The difference could be attributed to the populations tested, in this survey all participants were university students and the majority resided in cities, contrary to the population in the 2008 study where most participants had a high school education only, many were illiterate, and all resided in rural areas.

Good health-seeking behavior among university students was demonstrated in this study as well, since over 80% of participants would go to a doctor if they had TB symptoms and most would do it as soon as they suspect the symptoms were due to TB. Most participants also thought healthcare workers are a preferred source of information on TB, which further emphasizes good health-seeking behavior and indicates trust in the medical system. Most of the studies investigating health-seeking behavior were done in TB patients and indicated a significant association with TB knowledge (30, 31). This study also demonstrated a weak positive yet significant correlation between TB knowledge scores and both, attitude, and practice scores, similar to a study that was conducted on healthcare workers in Saudi Arabia (11).

Although the practice score was satisfactory in this study, it was found that around 41.0% of participants were not sure or did not agree that a mask is effective in preventing airborne diseases, which reached 52.5% in non-healthcare students. This could be addressed through education on the role of masks and when to use them. Several guidelines including those adopted by the WHO (32) and Center for Disease Control (CDC) (33) advocate for the use of respiratory protection when dealing with TB patients and especially when there is a risk of inhaling Mtb containing aerosols by a healthcare worker, although definitive practices such as the type of mask and when to wear it differs between guidelines and would require further evidence (34). A similar study conducted in Iran showed that around 86% agreed that wearing face masks is necessary for the examination of all patients, in contrast to 67.6% of healthcare students in this study, the higher percentage could be attributed to the settings of the study, which involved medical students in their last year of studies (28).

Only 11.5% of participants were aware that TB diagnosis and treatment are free in Jordan. This is very similar to another study conducted among a mainly uneducated population in the urban slums in Nigeria (35), which implies that treatment centers in Jordan should advertise treatment options more, especially since (85.9%) of participants wanted to learn more about TB, and a large percentage (73.6%) felt they were not well informed on TB. The distribution of TB knowledge could be done using new media outlets such as social media, which most participants (79.2%) believed was the most effective in reaching their peers, in agreement with a recent study from Oman that emphasized the role of digital platforms in TB knowledge dissemination (36).

This study has some limitations, for example, it was not possible to determine the actual response rate given the way the questionnaire was distributed, which took place through student communication channels online. Secondly, since this study involved several universities, we had to use an open platform for online forms and did our best to ensure that the survey reached only the population that was targeted, this was done by using locked communication channels accessible only to students with verified email addresses, nevertheless, the possibility of sending a link to a non-student outside those channels to complete the survey cannot be disregarded. But we believe that the detailed instructions at the start of the questionnaire (which emphasize that the questionnaire is meant only for students) and having to complete all answers across several pages to submit the form, make it unlikely that a substantial number of non-students, if any, have completed this form. Another limitation was that students who were informed about the topic of TB and pulmonary infections in general, were more likely to respond to the questionnaire, which could have introduced a sampling bias as alluded to earlier.

In conclusion, this study was the first to measure and demonstrate satisfactory KAP toward TB among Jordanian university students. The study underlined the need to promote knowledge on TB treatment and its availability, as well as TB transmission routes, especially among students from non-healthcare related fields. It also showed good health-seeking behavior in relation to TB but indicated a worrying trend of doubting the benefit of mask wearing among university students. Investigating the KAP toward TB among risk groups and refugee populations in Jordan is a logical next step in curtailing disease burden in the future.

## Data availability statement

The original contributions presented in the study are included in the article/Supplementary material, further inquiries can be directed to the corresponding author.

## Ethics statement

The studies involving human participants were reviewed and approved by the Institutional Review Board (IRB) at The University of Jordan (Ref. No. 19/2022/271). Decision No. (46-2022). The patients/participants provided their written informed consent to participate in this study.

## Author contributions

AA-H contributed to study conception, study design, data analysis, data interpretation, manuscript writing, and revision. AT contributed to study conception, acquisition and analysis of data, manuscript writing, and revision. YH contributed to study design, analysis of data, manuscript writing, and revision. AA-l, OA-l, and MA contributed to study design, acquisition of data, manuscript writing, and revision. FA, DA, and NA contributed to interpretation of data, manuscript writing, and revision. All authors contributed to the article and approved the submitted version.

## Conflict of interest

The authors declare that the research was conducted in the absence of any commercial or financial relationships that could be construed as a potential conflict of interest.

## Publisher's note

All claims expressed in this article are solely those of the authors and do not necessarily represent those of their affiliated organizations, or those of the publisher, the editors and the reviewers. Any product that may be evaluated in this article, or claim that may be made by its manufacturer, is not guaranteed or endorsed by the publisher.
