# Supplementary material for: Knowledge, attitudes, and practices toward tuberculosis among Jordanian university students
Source: Front Public Health. 2022 Nov 21;10:1055037. doi: 10.3389/fpubh.2022.1055037 (PMC9719926; doi:10.3389/fpubh.2022.1055037)
Supplement: Supplementary file 1 [file Data_Sheet_1.PDF]

Supplementary material

Knowledge, attitudes, and practices toward tuberculosis in Jordanian university students

Anas H. A. Abu-Humaidan[1*], Alaa Tarazi[2], Yazan Hamadneh[2], Ahmad Al-leimon[2], Obada Al-leimon[2], Mohammad Aljahalin[2], Fatima M. Ahmad[1], Dima Awajan[3], Nader Alaridah[1]

[1] Department of Pathology, Microbiology and Forensic Medicine, School of Medicine, The University of Jordan, Amman 11942, Jordan.

[2] School of Medicine, The University of Jordan, Amman 11942, Jordan

[3] Department of Clinical Pharmacy and Therapeutics, Applied Science Private University, Amman 11931, Jordan

*Address correspondence to:
Anas Abu-Humaidan M.D. Ph.D.
E-mail: A.abuhumaidan@ju.edu.jo
Tel. number: +962779227922

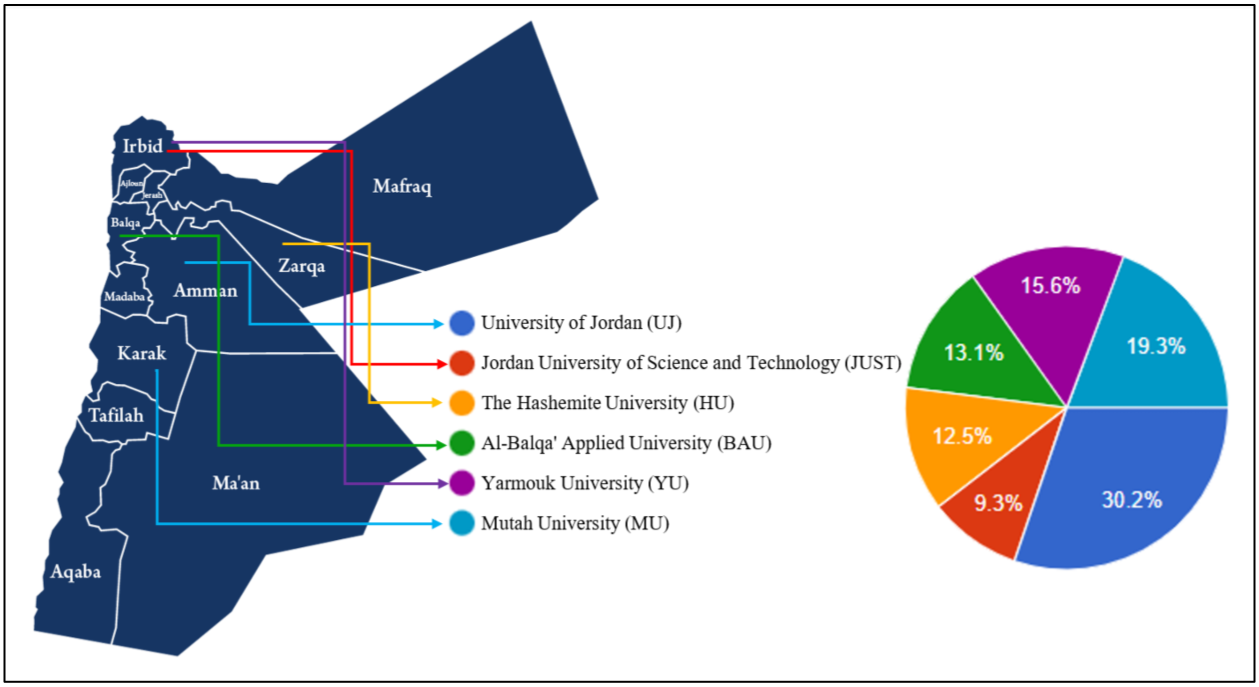

Supplementary figure 1. A representation of the percentage of students participating in the survey according to their university (pie chart to the right) and its location in Jordan (map to the left).

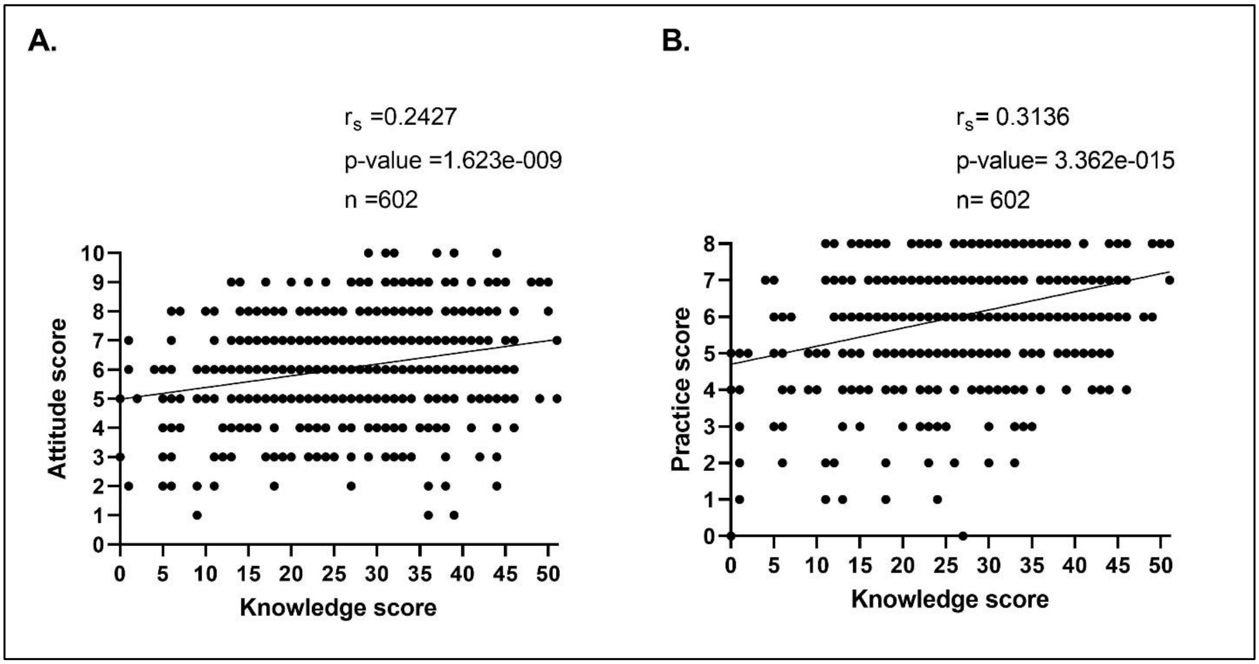

Supplementary figure 2. Scatter plots representing the correlation between (**A.**) knowledge and attitude scores, as well as (**B.**) knowledge and practice scores. Displayed on the plots are Spearman's correlation coefficient (r_s), p-values, and the number of participants (n).
